# Identification of lncRNA and mRNA regulatory networks associated with gastric cancer progression

**DOI:** 10.3389/fonc.2023.1140460

**Published:** 2023-03-06

**Authors:** Ke-kang Sun, Chao Zu, Xiao-yang Wu, Qing-hua Wang, Peng Hua, Yi-fang Zhang, Xiao-jun Shen, Yong-you Wu

**Affiliations:** ^1^ Department of Gastrointestinal Surgery, The Second Affiliated Hospital of Soochow University, Suzhou, Jiangsu, China; ^2^ Department of Gastrointestinal Surgery, Affiliated Kunshan Hospital to Jiangsu University, Suzhou, Jiangsu, China; ^3^ Department of Gastrointestinal Surgery, Sihong Hospital, Suqian, Jiangsu, China

**Keywords:** long non-coding RNAs, gastric cancer, expression profiling, lymph node metastasis, local invasion

## Abstract

Gastric cancer is a tumor type characterized by lymph node metastasis and the invasion of local tissues. There is thus a critical need to clarify the molecular mechanisms governing gastric cancer onset and progression to guide the treatment of this disease. Long non-coding RNAs and mRNA expression profiles associated with early and local advanced gastric cancer were examined through microarray analyses, with GO and KEGG analyses being employed as a means of exploring the functional roles of those long non-coding RNAs and mRNAs that were differentially expressed in gastric cancer. In total, 1005 and 1831 lncRNAs and mRNAs, respectively, were found to be differentially expressed between early and local advanced gastric cancer. GO and KEGG analyses revealed several pathways and processes that were dysregulated, including the RNA transport, ECM-receptor interaction, and mRNA splicing pathways. In co-expression networks, E2F1, E2F4, and STAT2 were identified as key transcriptional regulators of these processes. Moreover, thrombospondin-2 was confirmed as being expressed at high levels in more advanced gastric cancer by both the GEO and TCGA databases. RNA-sequencing analyses of SGC-790 cells transfected to express thrombospondin-2 further revealed this gene to enhance NF-kB and TNF pathway signaling activity. These results offer insight into gastric cancer-related regulatory networks and suggest thrombospondin-2 to be an important oncogene that drives the progression of this deadly cancer type.

## 1 Introduction

Gastric cancer (GC) is among the deadliest cancers in the world, and affected patients are broadly grouped into those with early and advanced disease. Early GC is restricted to the mucosal and submucosal layers irrespective of lymph node status, and can be effectively treated *via* surgical resection in many cases. In contrast, individuals with advanced disease exhibit a poor prognosis (1). Moreover, as there are few reliable symptoms or diagnostic biomarkers, a majority of patients are only diagnosed when the disease is already in an advanced stage. The mechanisms that govern GC progression are highly complex and tied to the aberrant expression and activity of a variety of cancer-associated genes (2). It is thus vital that these mechanisms be unraveled so as to guide the identification of novel biomarkers capable of aiding in the diagnosis and treatment of GC. In previous reports, mRNA expression-based approaches to detecting cancers at various stages of progression have been conducted (3).

Long non-coding RNA (lncRNAs) are over 200 nucleotides in length and regulate a diverse array of tumor-associated processes at the transcription and post-transcriptional levels, including epigenetic modifications, cellular differentiation, and cell cycle progression. Through chromosomal looping, for example, certain lncRNAs can drive chromatin rearrangement and thus affect transcription factor (TF) binding so as to alter transcriptional activity (4). A growing body of evidence supports the dysregulation of lncRNA expression in many malignancies including colorectal, breast, and renal cancer (5–7). While there have been some previous efforts to profile patterns of lncRNA expression associated with GC, relatively little remains known regarding how these patterns changes with disease progression. The present study was thus developed to compare lncRNA and mRNA expression patterns between early and local advanced GC patients. By constructing a co-expression network incorporating those mRNAs and lncRNAs that were differentially expressed, key signaling regulators and pathways were identified so as to clarify critical progression-associated genes.

## 2 Materials and methods

### 2.1 Tissue samples

In total, 26 paired primary GC tumor tissue samples and paracancerous tissue samples were obtained between February and August 2017. These patients included 13 with stage T1N0M0 and 13 with stage T4N3M0 disease, none of whom had undergone preoperative radiotherapy or chemotherapy. These patients either underwent radical distal gastrectomy or total gastrectomy, with pathological TNM staging having been assessed by three experienced pathologists in accordance with the National Comprehensive Cancer Network’s Clinical Practice Guidelines.

### 2.2 RNA extraction, microarray profiling and functional enrichment analyses

After isolation, GC patient tissues were snap-frozen with liquid nitrogen followed by storage at -80°C. OE Bioinformatics Technology Co., Ltd. (Shanghai, China) performed RNA extraction and all microarray profiling analyses, with samples being labeled, hybridized, and washed based on provided directions as detailed in prior studies (8).

### 2.3 Construction of the co-expression network

Potential correlative relationships between lncRNA co-expressed protein-coding genes and TF targets were assessed through Pearson correlation analyses. Hypergeometric distributions were used to assess GO term and pathway enrichment for coding genes exhibiting high coefficients, with the top 200 predicted relationships among differentially expressed lncRNAs and functional prediction terms being selected using Q-values and frequency counts, which was described in in prior studies (8).

### 2.4 Real-time quantitative reverse transcription-PCR

An RNeasy Mini Kit (217184, Qiagen, CA, USA) was used to extract RNA based on provided direction. Following analyses of RNA integrity and purity, a superscript III platinum kit (R250-01, Invitrogen) was used based on provided directions to prepare cDNA. SYBR Green I (CS7561, Invitrogen) was then used to conduct qPCR analyses with the ABIPrism 7500 instrument (Applied Biosystems) with the following settings: 40 cycles of 95°C for 10 s, 60°C for 30 s, and 70°C for 45 s. Relative gene expression was assessed based upon comparative threshold cycle (CT) values *via* the 2(^−ΔΔCt^) method, with GAPDH having been used for normalization. Primers were designed in accordance with target gene DNA sequences. The sequences of the primers used were as follows:

MUC6-F: 5′- AATTGTGATCTCTCAGGACGAG-3′
MUC6-R: 5′- CCTGAAGACCGTGATGTTGC-3′
THBS2-F: 5′- GACACGCTGGATCTCACCTAC-3′
THBS2-R: 5′- GAAGCTGTCTATGAGGTCGCA-3′
SOSTDC1-F: 5′- CCTAACTGGATTGGAGGAGGCT-3′
SOSTDC1-R: 5′- TCTGGGTACGGGTTTTGTCATT-3′
TLR5-F: 5′- CACGGAAGGTTGTGATGAA-3′
TLR5-R: 5′- GAGTGTCCAGGTGTTTGAG-3′
COL8A1-F: 5′- AGAACTACAACCCGCAGAC-3′
COL8A1-R: 5′- TTGAATAGAGCAACCCACA-3′
MT1G -F: 5′- AAGTGCAAAGAGTGCAAATGC-3′
MT1G -R: 5′- AGCAAAGGGGTCAAGATTGTAG-3′
GAPDH-F: 5′-CGACATGGAGAAAATCTGGCAC-3′
GAPDH-R: 5′-GATAGCACAGCCTGGATAGCAA-3′

### 2.5 Cell culture and transfection

SGC-790 cells were obtained from the Institute of Biochemistry and Cell Biology of the Chinese Academy of Science (Shanghai, China) and were cultured in DMEM containing 10% FBS (Invitrogen, CA, USA) containing penicillin/streptomycin (Invitrogen) in a 5% CO2 incubator at 37°C. Full-length thrombospondin-2 (THBS2) cDNA was prepared *via* PCR with specific primers selected based on the GenBank THBS2 reference sequence, with this cDNA then being cloned into the pcDNA3.0 eukaryotic expression vector (Genechem, Shanghai, China). Prior to transfection, SGC-790 cells were plated overnight in 4 cm dishes, followed by transfection with appropriate constructs using Lipofectamine 3000 (Invitrogen).

### 2.6 RNA-seq library preparation and sequencing

Trizol was used to extract total RNA based on provided directions, after which a NanoDrop 2000 spectrophotometer (Thermo Scientific, USA) was used to measure RNA concentrations and purity, while an Agilent 2100 Bioanalyzer (Agilent Technologies, CA, USA) was utilized to measure the integrity of isolated RNA. A TruSeq Stranded mRNA LT Sample Prep Kit (Illumina, CA, USA) was then used based on provided directions to prepare a sequencing library. All sequencing and analyses were performed by OE Bioinformatics Technology Co., Ltd. (Shanghai, China).

An Illumina HiSeq X Ten instrument was used to conduct 150 bp paired-end sequencing. Raw fastq data filed were initially processed with Trimmomatic, and clean reads were obtained *via* the removal of those reads considered of low quality. These clean reads were then aligned to the GRCh38 human reference genome with HISAT2, after which Cufflinks was used to calculate FPKM values for individual genes, while HTSeq-count was used to generate read counts. R was used to identify differentially expressed transcripts (P<0.05 and FC>2 or FC<0.5). Hierarchical clustering analyses of identified differentially expressed transcripts were then performed, after which GO and KEGG enrichment analyses of differentially expressed genes were conducted based upon hypergeometric distributions with R.

### 2.7 Statistical analysis

Data are means ± standard deviations, and were compared *via* Student’s t-tests using SPSS 19.0. P<0.05 was the threshold of significance.

## 3 Results

### 3.1 Analysis of mRNA and lncRNA dysregulation between early and local advanced GC

**Figure 1:**
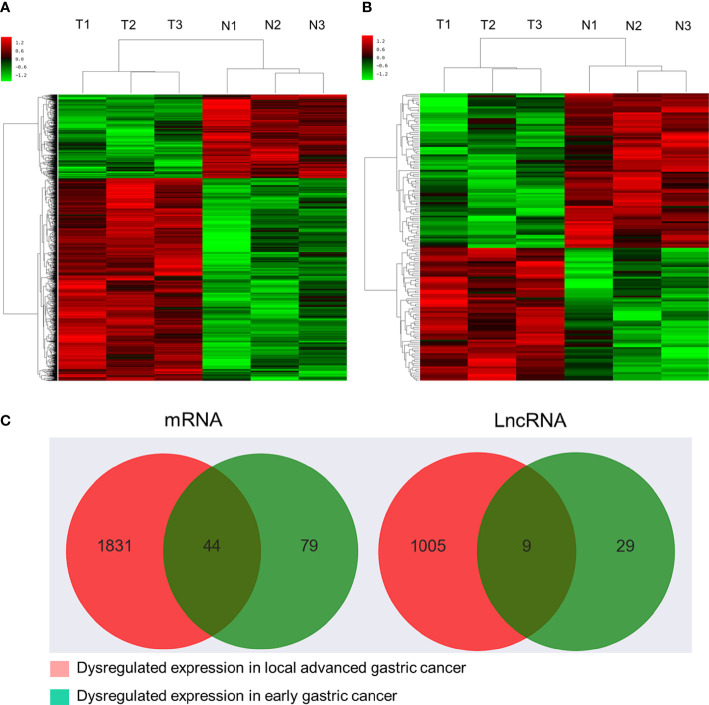
Microarray-based profiling of mRNAs and lncRNAs differentially expressed between GC tumors and paracancerous tissues. **(A)** Hierarchical clustering heatmap of transcripts differentially expressed between local advanced GC and paracancerous tissues. **(B)** Hierarchical clustering heatmap of transcripts differentially expressed between early GC and paracancerous tissues. **(C)** Venn diagram analysis of lncRNAs and mRNAs differentially expressed between locally advanced GC vs. paracancerous tissues and early GC vs. paracancerous tissues.

Initial global transcriptomic analyses identified 79,404 lncRNAs and 37,549 mRNAs, which were grouped into hierarchical clustering heat maps comparing local advanced and early GC tumor tissues to matched paracancerous healthy tissues (**Figures 1A, B**). In total, Venn diagram analyses revealed 1,005 dysregulated lncRNAs (516 upregulated, 489 downregulated) as well as 1,831 dysregulated mRNAs (1,277 upregulated, 554 downregulated) when comparing the difference set between local advanced and early GC (**Figure 1C**). The top 20 most dysregulated lncRNAs in local advanced disease identified *via* this approach were compiled in **Table 1**, with NONHSAT214974 and NONHSAT214368 respectively having been identified as the most downregulated and most upregulated lncRNAs. the most up-regulated. The top 20 most dysregulated mRNAs were similarly compiled in **Table 2**.

**Table 1:** Top 20 dysregulated lncRNA between gastric cancer with and without lymph node metastasis.

Target ID	FC (abs)	P	Regulation
NONHSAT214974	174	0.003	down
ENST00000482529	153	0.002	down
lnc-ADNP-1:1	118	0.004	down
ENST00000556770	112	0.002	down
lnc-C10orf103-5:1	73	0.001	down
lnc-SUN5-2:1	65	0.004	down
NR_033343	59	0.008	down
NONHSAT186948	57	0.002	down
lnc-B3GAT1-4:1	56	0.002	down
NONHSAT017372	56	0.044	down
NR_002919	56	0.007	down
T191147	54	0.005	down
lnc-RP11-116D17	49	0.021	down
NONHSAT187473	45	0.004	down
NONHSAT197042	44	0.003	down
NONHSAT214368	43	0.003	up
ENST00000242208	42	0.001	up
XM_005266617	42	0.004	down
NONHSAT177338	39	0.014	down
AL713743	35	0.003	down

**Table 2:** Top 20 dysregulated mRNA between gastric cancer with and without lymph node metastasis.

Gene Symbol	Target ID	FC (abs)	P	Regulation
GKN2	NM_182536	860	0.001	down
MUC6	NM_005961	232	0.028	down
MUC5AC	XM_006709945	141	0.004	down
CA9	NM_001216	93	0.004	down
PSCA	NM_005672	87	0.026	down
DISP1	ENST00000360254	75	0.002	down
KIAA1324	NM_020775	70	0.019	down
C6orf58	NM_001010905	66	0.003	down
REG1A	NM_002909	64	0.048	down
UMODL1	ENST00000491559	48	0.001	down
CPA2	NM_001869	44	0.005	down
AKR1C1	NM_001353	39	0.001	down
THBS2	NM_003247	30	0.002	up
C4orf17	NM_032149	30	0.003	down
SOSTDC1	NM_015464	29	0.029	down
TLR5	NM_003268	29	0.002	down
FLG	NM_002016	25	0.005	down
COL8A1	NM_001850	24	0.011	up
DKK1	NM_012242	24	0.004	up
MT1G	NM_001301267	22	0.002	down

### 3.2 Functional enrichment analyses of dysregulated lncRNAs and mRNAs

**Figure 2:**
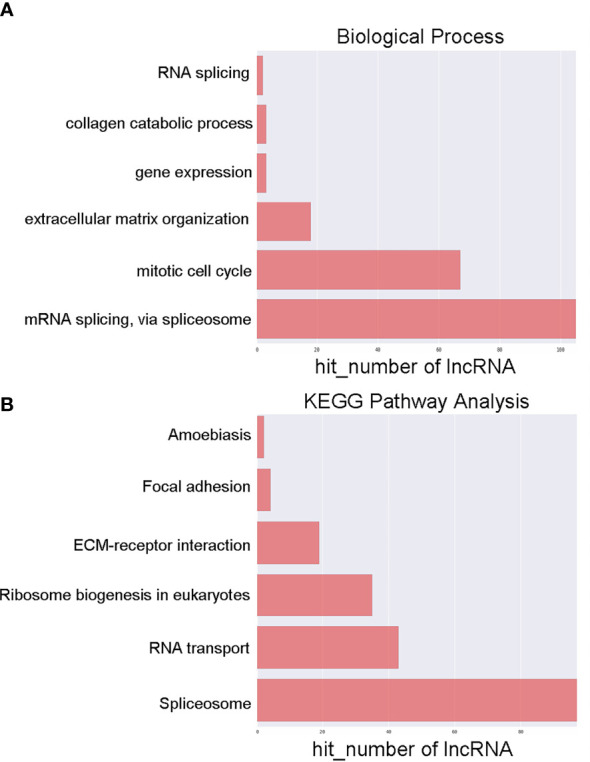
GO and KEGG pathway analyses of lncRNAs differentially expressed between early-stage and locally advanced GC. **(A)** The top 6 biological process GO terms corresponding to dysregulated lncRNAs. **(B)** The top 6 KEGG terms corresponding to dysregulated lncRNAs. Annotated terms and numbers of genes associated with those terms are respectively shown on the y-axis and the x-axis.

**Figure 3:**
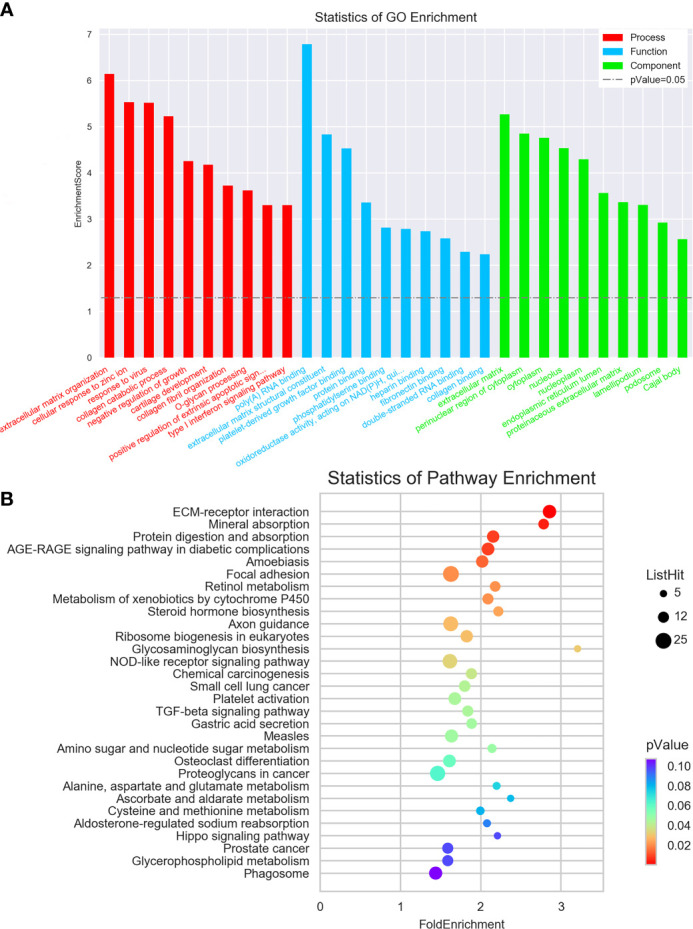
GO and KEGG pathway analyses of mRNAs differentially expressed between early-stage and locally advanced GC. **(A)** The top 10 GO terms in each category corresponding to dysregulated mRNAs. **(B)** The top 30 KEGG pathways enriched in mRNAs dysregulated in locally advanced GC.

To examine the possible functional roles of those lncRNAs found to be differentially expressed in GC, GO and KEGG enrichment analyses of the top 200 most differentially expressed lncRNAs were performed. These lncRNAs were found to be enriched in GO terms including mRNA splicing, mitotic cell cycle, and extracellular matrix organization (**Figure 2A**), as well as in corresponding KEGG pathways including the spliceosome, RNA transport, ribosome biogenesis in eukaryotes. and ECM-receptor interaction pathways (**Figure 2B**). Similarly, differentially expressed mRNAs were enriched in GO terms including extracellular matrix organization, cellular response to zinc ions, poly(A) RNA binding, extracellular matrix structural constituent, extracellular matrix, and perinuclear region of the cytoplasm (**Figure 3A**), as well as in KEGG pathways including the ECM-receptor interaction and mineral absorption pathways (**Figure 3B**).

### 3.3 Co-expression network analyses

**Figure 4:**
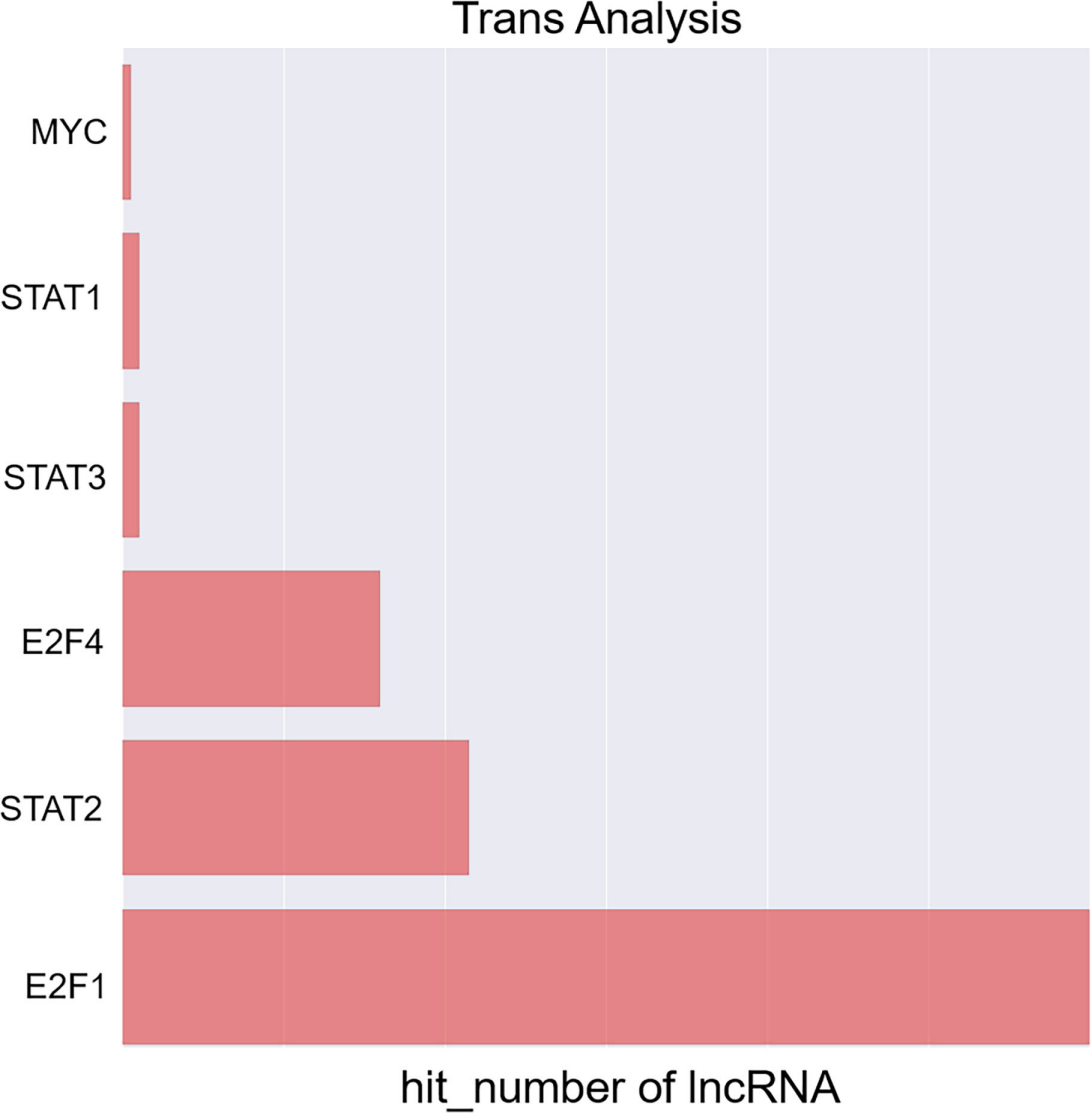
Profiling of transcription factors associated with lncRNAs most significantly dysregulated between local advanced GC and early GC.

**Figure 5:**
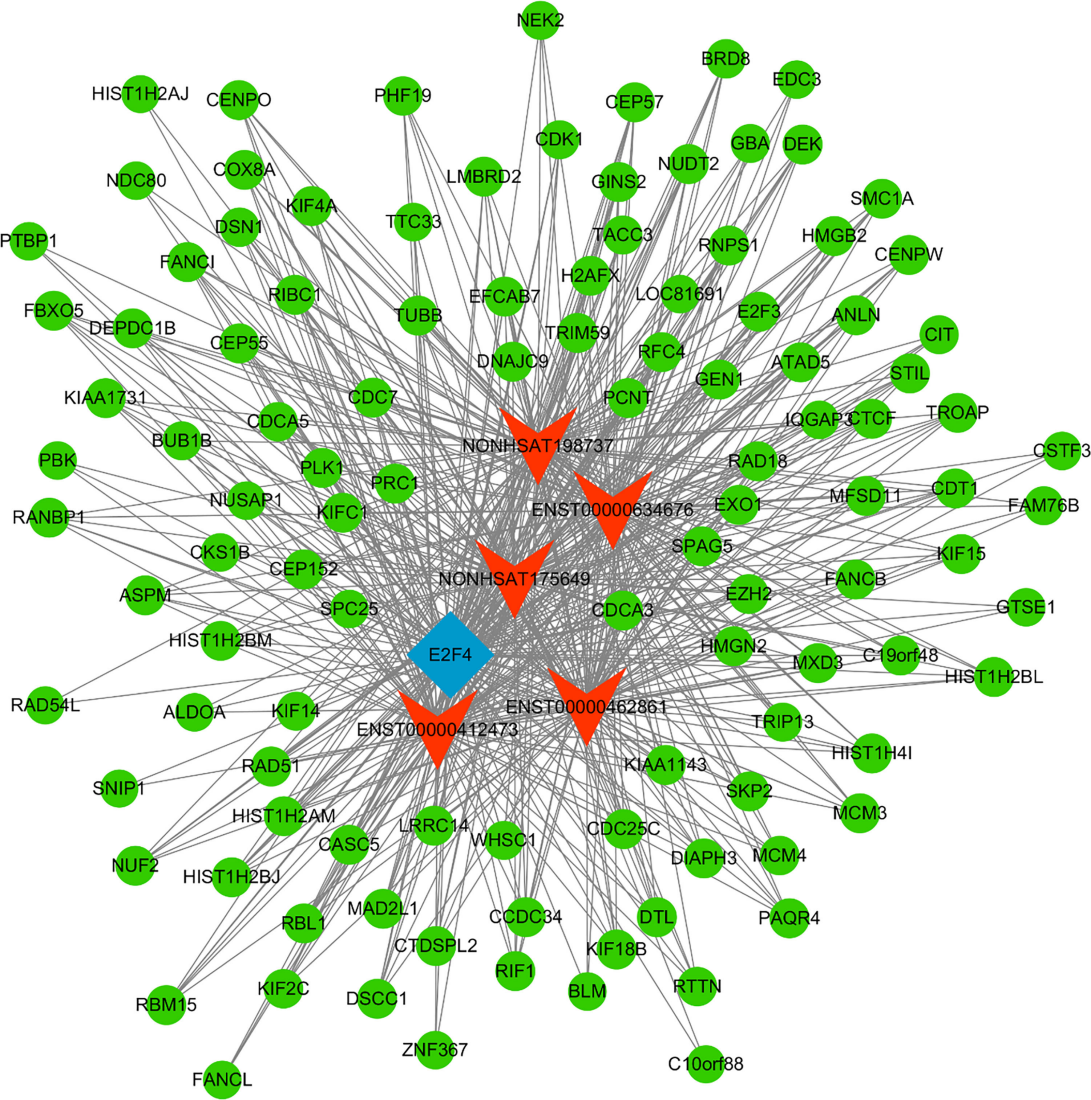
TF-lncRNA-mRNA network analysis. mRNAs, lncRNAs, and TFs are respectively represented with green, red, and blue nodes.

Next, a co-expression network was constructed incorporating those lncRNAs and mRNAs that were co-expressed (Pearson’s correlation coefficient ≥ 0.99). In an effort to identify the potential trans-regulatory functions of the lncRNAs within this network, those mRNAs that were co-expressed with lncRNAs and were known to be regulatory targets of specific TFs were further analyzed. This approach suggested that the identified lncRNAs may be associated with the regulatory activity of E2F1, E2F4, and STAT2 (**Figure 4**). In the constructed co-expression network, individual mRNAs and lncRNAs were correlated with anywhere between 1 and 10 lncRNAs (**Figure 5**), thus suggesting that the complex inter-regulatory relationships among these transcripts may be central to the process of GC progression.

### 3.4 qPCR-based validation of microarray results

**Figure 6:**
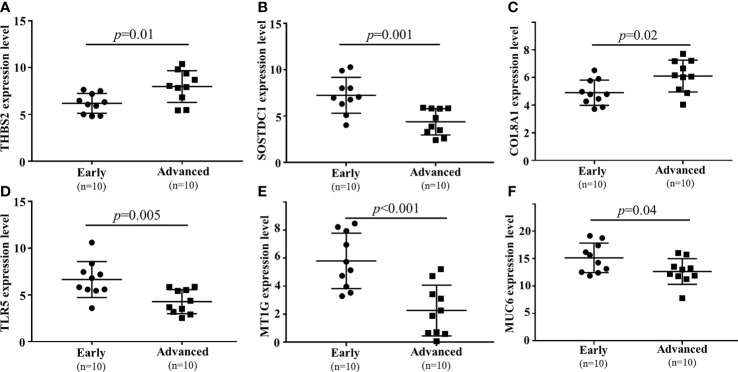
qPCR-based validation of 6 mRNAs (**(A)** THBS2, **(B)** SOSTDC1, **(C)** COL8A1, **(D)** TLR5, **(E)** MT1G, **(F)** MUC6) dysregulated between local advanced GC and early GC.

To confirm the results of the above analyses, a qPCR approach was next used to confirm the differential expression of six mRNAs. The expression level of lncRNAs had been verified in previous studies (8). In this analysis, the expression of six filtered mRNAs was verified by qRT-PCR in another 10 early GC and 10 local advanced GC patients. SOSTDC1, TLR5, MT1G, and MUC6 were downregulated in locally advanced tumor tissue samples, whereas COL8A1 and THBS2 were upregulated (P<0.05, **Figure 6**). These results were consistent with the finding from the microarray conducted above.

### 3.5 THBS2 is related to GC progression

**Figure 7:**
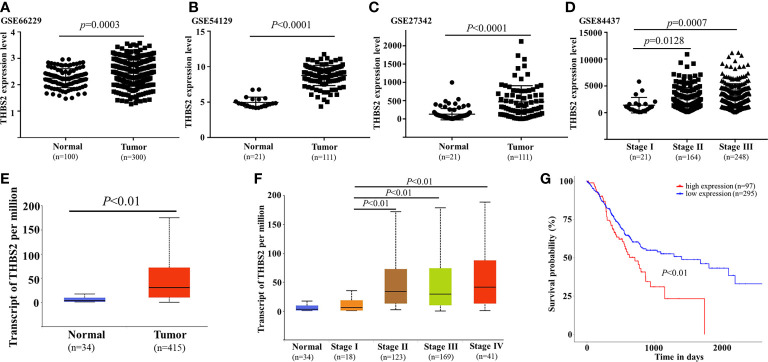
Analysis of THBS2 expression dynamics in GC. THBS2 mRNA levels were analyzed in the TCGA **(E)** and GEO databases (**(A)** GSE66229, **(B)** GSE54129, **(C)** GSE27342). THBS2 expression was compared across various stages of GC in the TCGA **(D)** and GEO **(F)** databases. Patient overall survival as a function of THBS2 expression was analyzed in the TCGA database **(G)**.

As it was identified as one of the most upregulated mRNAs associated with locally advanced GC, THBS2 was selected for further validation experiments. Analyses of the GEO and TCGA databases revealed the upregulation of THBS2 in GC tumor tissues relative to normal paracancerous tissues (TCGA: P<0.01; GSE66229: P=0.003; GSE54129: P<0.0001; GSE27342: P<0.0001; **Figures 7A–C, E**). THBS2 was also expressed at significantly higher levels in advanced tumors relative to those collected from patients with early-stage disease (**Figures 7D, F**). Kaplan-Meier analyses revealed that patients expressing high levels of THBS2 exhibited poorer overall survival within the TCGA cohort (P<0.01, **Figure 7G**). As such, THBS2 may be related to GC progression.

### 3.6 Identification of genes potentially regulated by THBS2

**Figure 8:**
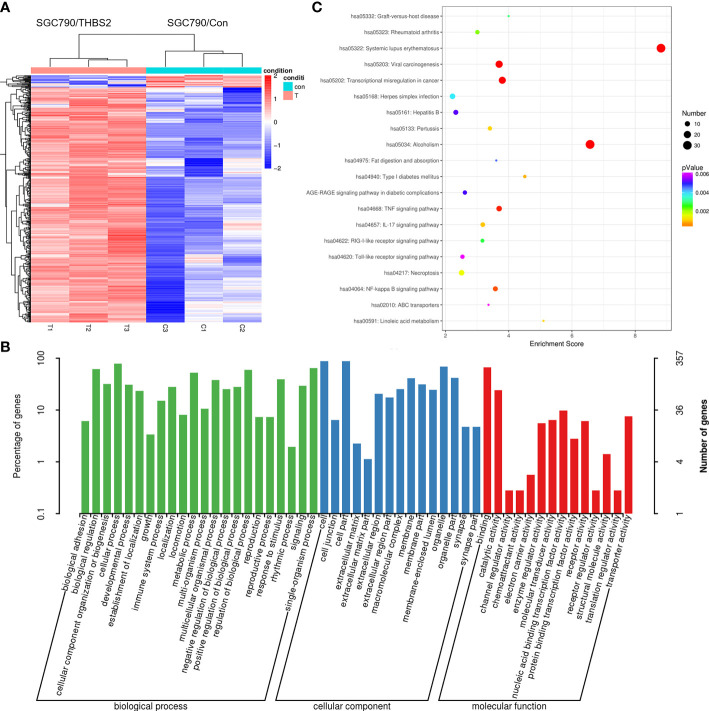
RNA-seq analysis of gene expression patterns in control and THBS2-overexpressing SGC-790 cells. **(A)** Dysregulated mRNAs are represented using a heatmap. **(B)** Top GO terms associated with mRNAs that were differentially expressed in SGC-790 cells overexpressing THBS2. **(C)** Top KEGG pathways associated with mRNAs that were differentially expressed in SGC-790 cells overexpressing THBS2.

Lastly, an RNA-seq approach was used to identify patterns of differential gene expression between SGC-790 cells that had been transfected with control and THBS2 overexpression constructs. In total, comparisons of these cells revealed 445 and 23 genes that were significantly up- and downregulated, respectively (FC>2, FC<0.5, P<0.05). These genes were further arranged into a hierarchical cluster heatmap (**Figure 8A**), and were subjected to GO and KEGG enrichment analyses revealing them to be enriched in GO terms including binding, transporter activity, extracellular matrix, and cell junction (**Figure 8B**). Moreover, they were enriched in the TNF signaling, transcriptional misregulation in cancer, and NF-kB signaling pathways (**Figure 8C**).

## 4 Discussion

High-throughput sequencing analyses are widely employed as tools to explore the genomic responses to particular drugs, disease states, and cancers. Here, a microarray approach was employed to generate co-expression networks composed of the core regulators of GC pathogenesis and progression. In total, 1,005 lncRNAs (516 upregulated, 489 downregulated) and 1,831 mRNAs (1,277 upregulated, 554 downregulated) were capable of effectively differentiating between early-stage and locally advanced GC, with these results being in line with a previous report (9). These progression-related genetic changes included several mRNAs and lncRNAs of known oncogenic relevance, thus highlighting a valuable foundation for future efforts to test GC progression-related targets (10, 11).

GO and KEGG pathway analyses were implemented as a means of exploring the functional roles of the transcripts found to be differentially expressed in the context of GC progression. Dysregulated lncRNAs were found to be enriched in GO biological process terms including mRNA splicing, mitotic cell cycle, and extracellular matrix organization, as well as in KEGG pathways including the spliceosome, RNA transport, ribosome biogenesis in eukaryotes, and ECM-receptor interaction pathways. These lncRNAs may therefore act as key regulators and viable targets associated with GC progression. Similarly, differentially expressed mRNAs were found to exhibit patterns of expression in line with those reported previously (3), and GO and KEGG pathways indicated that they may be related to extracellular matrix organization and other pathways relevant to GC progression such that they may be promising targets for therapeutic intervention. Moreover, a constructed TF-lncRNA-mRNA network highlighted E2F1, E2F4, and STAT2 as potentially important TFs associated with the regulatory activity of identified GC-related lncRNAs.

Thrombospondin-2 is a matrix glycoprotein that has been shown to regulate key activities including ECM remodeling, cellular adhesion, proliferation, and angiogenesis (12). The expression of THBS2 varies among cancers, and its precise relevance as a driver of tumor progression is still the subject of controversy (13–15). Here, THBS2 was the mRNA that was most upregulated in local advanced GC relative to early cases. Analyses of the GEO and TCGA databases similarly confirmed that GC tumors exhibited THBS2 upregulation relative to healthy paracancerous tissue, and that this gene was more significantly upregulated in tumors that were more advanced. To confirm the relevance of THBS2 as a functional regulator of tumor progression rather than an incidentally upregulated target, an RNA-seq analysis of GC cells overexpressing this gene was conducted, revealing that increased THBS2 expression was associated with enhanced TNF and NF-kB signaling pathway activity, in addition to being related to the transcriptional misregulation in cancer pathway. As such, THBS2 may function as a critical oncogene that regulates the progression of GC.

In conclusion, these analyses offer new microarray-based insights into the roles played by particular lncRNAs and mRNAs in the context of GC progression. Further analyses of these data have the potential to guide the further clarification of the mechanisms governing the progression of this deadly disease. Moreover, THBS2 was identified as a potential key regulator and therapeutic target associated with this cancer type. We will further verify the function and specific mechanism of THBS2 in GC cell metastasis and invasion through biological experiments. Furthermore, our ongoing investigations will seek to extend these data by identifying additional GC-related lncRNAs with the potential to serve as diagnostic or prognostic biomarkers related to GC progression.

## Data availability statement

The datasets presented in this study can be found in online repositories. The names of the repository/repositories and accession number(s) can be found in the article/supplementary material.

## Ethics statement

The studies involving human participants were reviewed and approved by The Ethics Committee of Jiangsu University. The patients/participants provided their written informed consent to participate in this study. Written informed consent was obtained from the individual(s) for the publication of any potentially identifiable images or data included in this article.

## Author contributions

K-KS and Y-YW contributed to conception and design of the study. K-KS organized the database CZ performed the statistical analysis. K-KS wrote the first draft of the manuscript. X-YW, Q-HW, PH, Y-FZ, and X-JS wrote sections of the manuscript. All authors contributed to the article and approved the submitted version.
